# Ashy Dermatosis and Lichen Planus Pigmentosus: The Histopathological Differences

**DOI:** 10.1155/2019/5829185

**Published:** 2019-10-28

**Authors:** Suthinee Rutnin, Siriorn Udompanich, Nathathai Pratumchart, Sarawin Harnchoowong, Vasanop Vachiramon

**Affiliations:** Division of Dermatology, Faculty of Medicine Ramathibodi Hospital, Mahidol University, Bangkok, Thailand

## Abstract

**Background:**

Ashy dermatosis (AD) and lichen planus pigmentosus (LPP) are both acquired macular pigmentation of uncertain aetiology. Despite the controversy surrounding their entities, recent global consensus has concluded that they are 2 different diseases with distinct clinical presentations. Nevertheless, there are limited data on their histopathological comparisons.

**Objective:**

To evaluate the differences in histopathological findings between AD and LPP.

**Methods:**

Electronic records and photographs of patients with the diagnosis of AD or LPP from January 2008 to December 2018 were retrospectively reviewed by a dermatologist. Patients were then classified into groups with AD and LPP, based on the clinical descriptions from the recent consensus. Those with history/clinical presentations suggestive of other causes of macular pigmentation were excluded. The histopathological diagnosis of AD and LPP was then reevaluated by a blinded dermatopathologist.

**Results:**

One hundred and twenty-four patients with acquired macular pigmentation were identified; 24 were excluded due to clinical history or photographs being inconsistent with AD or LPP. Of the remaining 100 patients, 71 had clinical findings consistent with LPP while 29 had AD. The prevalence of epidermal hyperkeratosis was significantly higher in LPP when compared to AD (33.8% vs. 0%, *p* < 0.001), as well as epidermal hypergranulosis (35.2% vs. 0%, *p* < 0.001), lichenoid dermatitis (49.3% vs. 7.1%, *p* < 0.001), perifollicular infiltration (47.9% vs.10.3%, *p* < 0.001), and perifollicular fibrosis (35.2% vs. 10.3%, *p*=0.01). In addition, the degree of pigmentary incontinence was more severe in LPP (21.1% vs. 3.5%, *p*=0.015). For AD, vacuolization of the epidermal basal cell layer was more common (96.4% vs. 77.5%, *p*=0.02).

**Conclusions:**

Although most cases of AD and LPP can be diagnosed clinically, in doubtful cases, histopathological findings of lichenoid dermatitis, epidermal hyperkeratosis/hypergranulosis, and moderate to severe pigmentary incontinence can help distinguish LPP from AD.

## 1. Introduction

Ashy dermatosis (AD) and lichen planus pigmentosus (LPP) are both macular pigmentation of uncertain aetiology, predominantly found in patients with skin phototypes III-IV [1]. Despite being asymptomatic, the hyperpigmentation can still cause significant distress to the patients and compromise their quality of life [2]. AD typically presents with insidious ill-defined bilateral slate-grey patches along the skin cleavage lines on the trunk, proximal extremities, and face [3, 4]. The term erythema dyschromicum perstans (EDP) can be synonymous with AD except for the presence of erythematous border in the active stage of EDP [5]. LPP are grey macules and patches with purplish hue, most commonly on the face, neck, and flexures, with varying patterns of hyperpigmentation and possible association with lichen planus and hepatitis C infection [6–8].

In the last decade, few studies have proposed that AD and LPP are the same disease on two ends of the spectrum, most likely due to the timing of diagnosis [9, 10]. Others believe they are 2 separate entities [11, 12], with entirely different pathomechanisms. A recent global consensus compiled by Kumarasinghe et al. has concluded that AD and LPP do have distinct clinical presentations but share similar features on histopathology, mainly interface dermatitis and pigmentary incontinence [5]. Limited studies have attempted to evaluate their histopathological differences to help differentiate the two conditions [12, 13]. Our study aims at evaluating the differences in histopathological findings between AD and LPP and exploring their clinical and histological correlations.

## 2. Materials and Methods

This retrospective observational analytic study was approved by Ramathibodi Hospital, Mahidol University Institutional Review Board (ID 09-60-45). Electronic records of patients with acquired macular pigmentation from the university-based hospital, Bangkok, Thailand, between January 1, 2008, and December 31, 2018, were collected. Only patients with available photographs consistent with AD or LPP and available biopsy specimen from the hyperpigmented lesion were included in the study. Patients with any medications that can cause lichenoid drug eruption, history suggesting pigmented contact dermatitis, with or without a positive patch test, or history of preceding erythematous lesions suggesting postinflammatory hyperpigmentation (PIH) were excluded.

**Figure 1:**
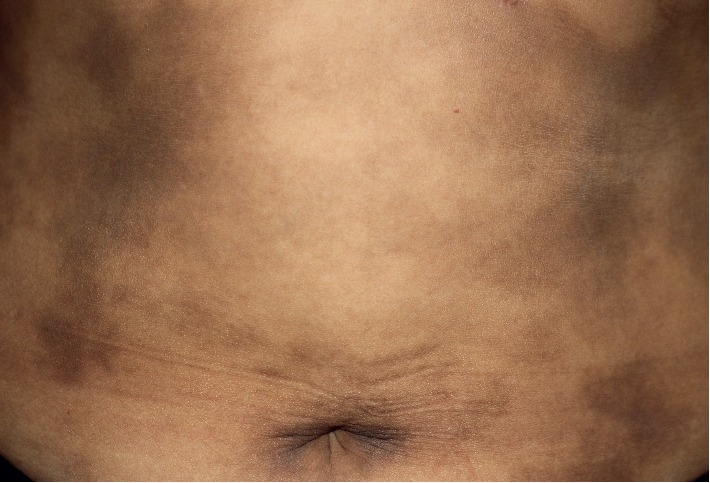
Typical characteristics of AD on clinical examination.

**Figure 2:**
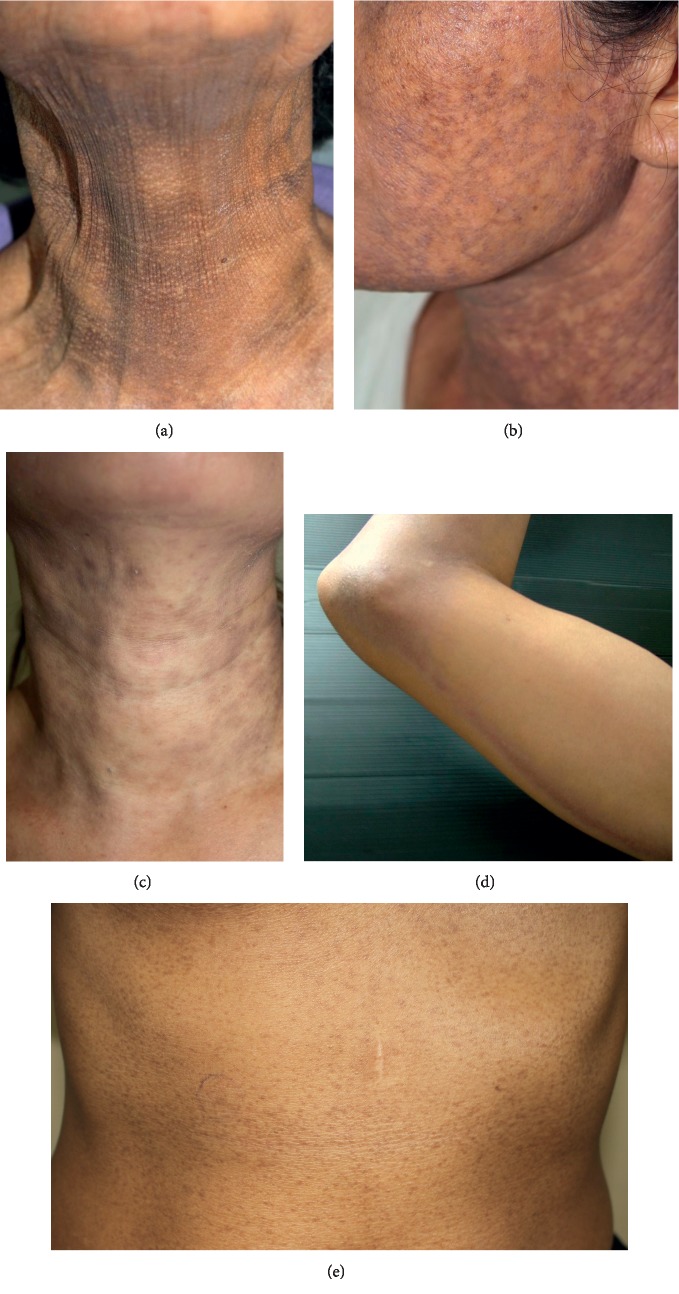
Different patterns of pigmentation in LPP: (a) diffuse, (b) reticular, (c) blotchy, (d) linear, and (e) perifollicular.

After exclusion, the clinical photographs were reviewed by a blinded dermatologist. Each patient was then classified as having either AD or LPP, based on the site, colour, morphology, and distribution of the lesion, as proposed by the recent global consensus [5]. According to the consensus, features that suggest AD were large hyperpigmented macules (>5 cm) with or without an erythematous border on the trunk (Figure 1) and features that suggest LPP were a combination of large macules (>5 cm) and small macules (0.5–5 cm) on the head, neck and flexural areas. Different patterns of pigmentation in LPP were documented, as shown in Figures 2(a)–2(e). Any ambiguous photographs or photographs consistent with hyperpigmentation from other causes were excluded.

After being allocated into groups with AD and LPP clinically, patients' demographic data, history of rash, and associated conditions were documented. All histopathological specimens were then reevaluated by one blinded dermatopathologist who was unaware of their history and clinical presentation. The histopathological assessment was performed from the epidermis down and included these following features: epidermal changes (atrophy, hyperkeratosis, and hypergranulosis), interface changes (basal vacuolization and lichenoid dermatitis), superficial perivascular infiltration (mild, moderate, and severe), perifollicular infiltration/fibrosis, perieccrine infiltration, and pigmentary incontinence. The degree of pigmentary incontinence was categorized into mild (fewer than 10 melanophages/HPF), moderate (10–20 melanophages/HPF), and severe (>20 melanophages/HPF) [10].

Statistical analysis was performed on Stata 14.0 (StataCorp LLC, College Station, TX). Chi-square and Fisher exact tests were used to evaluate the association between the diagnosis made from photographs and each histopathological feature (categorical outcomes). For continuous data, such as disease duration, Mann–Whitney *U* and Kruskal–Wallis tests were conducted. *p* value less than 0.05 was considered significant.

## 3. Results

**Figure 3:**
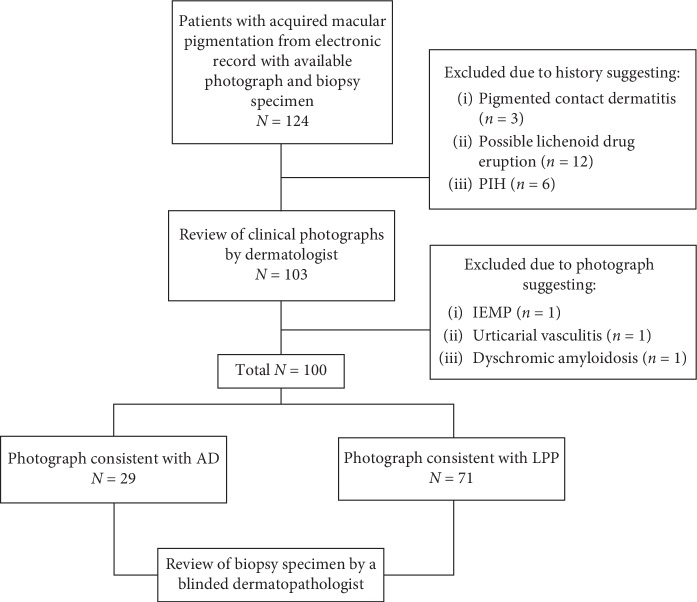
Protocol flowchart. AD, ashy dermatosis; IEMP, idiopathic eruptive macular pigmentation; LPP, lichen planus pigmentosus; PIH, postinflammatory hyperpigmentation.

One hundred and twenty-four acquired macular pigmentation cases were gathered from the electronic records; 21 were excluded due to history consistent with pigmented contact dermatitis (*n* = 3), lichenoid drug eruption (*n* = 12), and PIH (*n* = 6). Three patients with photographs suggestive of hyperpigmentation from other causes, including idiopathic eruptive macular pigmentation (IEMP), urticarial vasculitis, and dyschromic amyloidosis were also excluded. Ultimately, biopsies from clinically diagnosed 29 AD and 71 LPP cases were sent for a histopathological review (Figure 3).

### 3.1. Demographic Data

Table 1 summarizes the baseline characteristics, history, and associated conditions of patients in each group. The mean age at diagnosis was significantly higher in the LPP group when compared to the AD group (49.6 vs. 42.4 years, *p*=0.03). However, the gender and Fitzpatrick skin type were not significantly different between the two conditions. Although most patients were asymptomatic, pruritus was more commonly observed in the LPP group compared to the AD group (31.0% vs. 13.8%, *p*=0.08) while a burning sensation was described in few patients from both groups. Associations with other conditions were noted in the LPP group as follows: concurrent oral lichen planus in 1 patient; lichen planopilaris in 6 patients, all in the form of frontal fibrosing alopecia (LPP preceded FFA in 2 cases, occurred at the same time in 2 cases, FFA preceded LPP in 1 case, and unknown onset in 1 case); and viral hepatitis infection in 6 patients (hepatitis B infection in 4 cases and hepatitis C infection in 2 cases). In the AD group, there were no associations with any other conditions except for 3 patients who were documented to have coexisting hypothyroidism.

**Figure 4:**
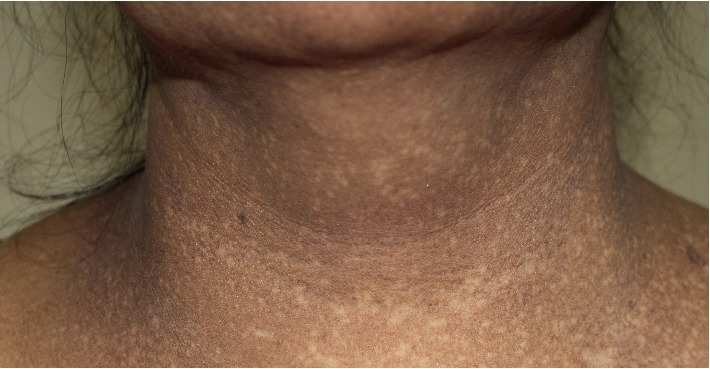
Guttate hypopigmentation in LPP.

Details of the clinical characteristics of AD and LPP in this study are listed in Table 2. The majority of AD lesions were slate-grey (58.6%) and brown-grey (34.5%), while in LPP they were brown-grey (62%) and purplish-grey (33.8%). The erythematous ring was observed in only one AD patient. Both conditions mostly presented with bilateral ill-defined patches, which were more likely to be larger than 5 cm in AD. The most common pattern of pigmentation in LPP was reticular (46.5%), followed by diffuse (31.0%) and blotchy (19.7%). In terms of linear and perifollicular pattern, each was found in only one patient. Interestingly, guttate hypopigmentation scattered within the hyperpigmented patches was found in a small proportion of LPP patients (8.5%) (Figure 4). In these patients, neither features of scleroderma nor hydroquinone application has been noted. The most common areas of involvement in AD were the face/neck (58.6%), back (55.2%), and abdomen (48.3%) while a large proportion of LPP patients had lesions on the face/neck (80.3%), flexors (64.8%), and upper extremities (40.9%). In addition, there was a slightly higher predilection for sun-exposed areas in LPP.

### 3.2. Histopathological Differences

Our histopathological review confirmed that certain histopathological features are frequently observed in both groups, such as the basal vacuolization along the dermoepidermal junction (DEJ), superficial perivascular lymphocytic infiltration, and presence of melanophages in the upper dermis. The interface changes mostly occurred in focal areas in both conditions. However, detailed histopathological examination revealed that some features may point towards one condition more than the other (Table 3).

**Figure 5:**
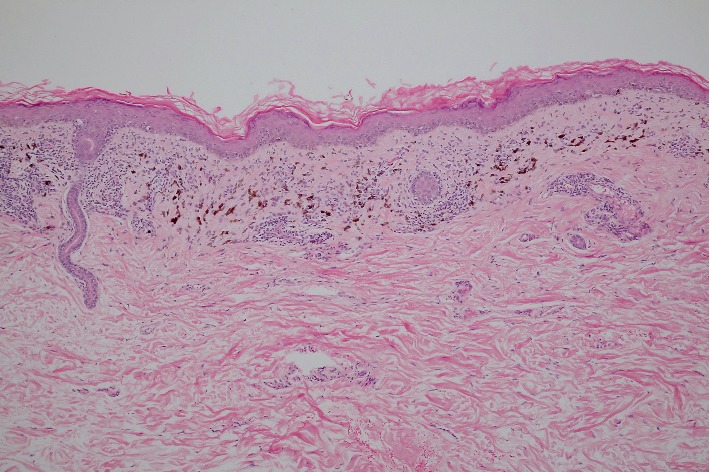
Histopathology of LPP patient demonstrating epidermal hyperkeratosis and focal hypergranulosis, focal lichenoid dermatitis, and moderate superficial perivascular lymphocytic infiltration with numerous dermal melanophages (hematoxylin-eosin stain, original magnification ×100).

**Figure 6:**
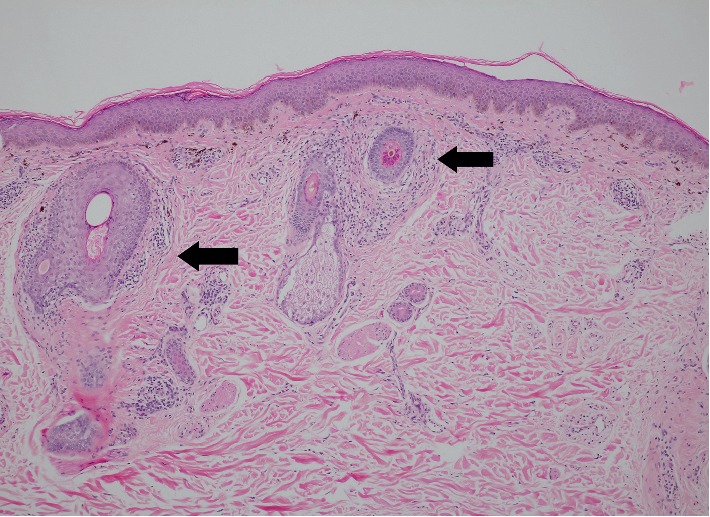
Perifollicular lymphocytic infiltration and fibrosis (arrow) in LPP (hematoxylin-eosin stain, original magnification ×100).

In LPP, the epidermal changes were more pronounced. Epidermal hypergranulosis and hyperkeratosis (Figure 5) occurred in 35.2% and 33.8% of LPP patients, respectively, while none was observed in AD. Lichenoid dermatitis, mostly focal, was present in half of the patients with LPP and was significantly more common in LPP when compared to AD (*p* < 0.001). The intensity of superficial perivascular lymphocytic infiltration and the intensity of pigmentary incontinence were also significantly more severe in LPP (*p*=0.04 and 0.015, respectively). Another striking feature in LPP was the perifollicular involvement (Figure 6). Perifollicular infiltration was found in up to 47.9% of patients, while 35.2% developed some degree of perifollicular fibrosis. Both features were significantly more common in LPP (*p* < 0.001 for infiltration and *p*=0.01 for fibrosis). Furthermore, perieccrine infiltration was only observed in LPP, but only in a small number of patients.

**Figure 7:**
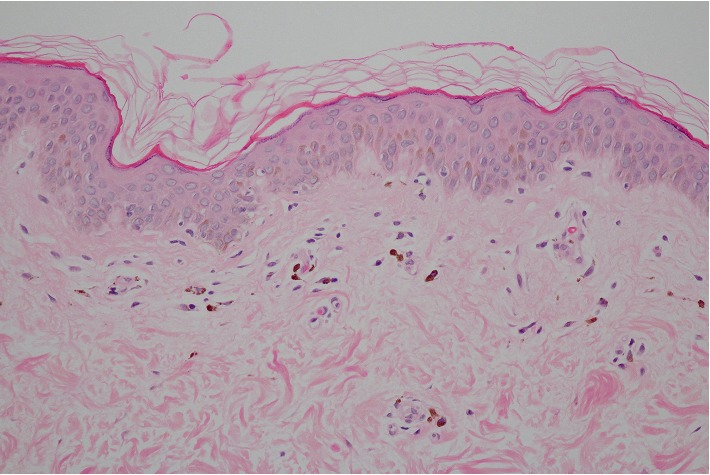
Histopathology of AD patient showing a normal epidermis, focal basal vacuolization along the DEJ, sparse superficial perivascular lymphocytic infiltration, and mild melanophage deposition (hematoxylin-eosin stain, original magnification ×100).

When compared to LPP, epidermal atrophy was slightly more frequent in AD (24.1% vs. 18.3%, *p*=0.51). Overall, the degrees of inflammation and pigmentary incontinence were less severe (Figure 7). The predominant interface change was focal basal vacuolization along the DEJ, which was present in almost all AD patients (96.4%). In contrast to LPP, lichenoid dermatitis and perifollicular infiltration/fibrosis were only detected in a few patients.

### 3.3. Clinicopathological Correlation

Some histopathological features were reflected on their clinical presentation. The presence of lichenoid dermatitis and severe melanophage deposition both correlated with purplish-grey colour on clinical examination (*p* value = 0.026 and 0.043, respectively). There was no correlation between any histopathological changes and certain pattern of pigmentation in LPP. In addition, the presence of epidermal changes, dense inflammatory infiltrate, interface changes, or severity of melanophage deposition did not correlate with disease duration.

### 3.4. Treatment Outcomes

The most popular treatment modalities were topical corticosteroids, depigmenting agents, and moisturizers. Systemic agents and pigment lasers were rarely used, with a disappointing outcome. No particular treatment was associated with a significant improvement in the lesion. A majority of patients (44.8% of AD and 47.9% of LPP) achieved partial resolution at an average of 21.4 weeks for AD and 15.5 weeks for LPP. A complete resolution was only reported in one patient with LPP, after applying topical corticosteroid and vitamin A for 3 years.

## 4. Discussion

The term ashy dermatosis (AD) was first described in 1967 by Ramirez as asymptomatic macular lesion with various shade of grey pigmentation [14]. Identical lesions, but with raised erythematous borders, were later termed EDP [15]. Nowadays, AD and EDP are considered synonymous. In 1974, Bhutani et al. reported slate-blue to steel-grey hyperpigmentation in 40 Indian patients with possible relation to lichen planus and thus coined the term LPP [16]. Due to their overlapping clinical features, there have been some controversies regarding their identities. Recently, a global consensus has stated that AD and LPP are two distinct conditions with similar histopathological features [5].

As mentioned earlier, two prior studies compared histopathological features of AD and LPP and concluded that they are indistinguishable on histopathology [12, 13]. However, in both studies, the criteria for AD/LPP diagnosis were made prior to the global consensus [5] and histopathological evaluations were performed in a limited number of specimens. To the best of our knowledge, we were the first group to review each case individually from photographs and classify the patients into groups with AD and LPP, based on the clinical features which were agreed upon in the recent consensus [5]. We found that LPP is significantly associated with the histopathological features of epidermal hyperkeratosis and hypergranulosis, focal lichenoid dermatitis, perifollicular involvement, moderate to severe inflammatory cell infiltration, and pigmentary incontinence. In contrast, the histopathological changes in AD were more subtle, with predominant focal basal vacuolization along the DEJ, mild inflammatory infiltrate, and melanophage deposition.

The histopathological features found in this study may help explain the pathomechanism of each disease. LPP had long been considered a variant of lichen planus, with similar pathogenesis mediated by T lymphocytes where CD8+ T cells recognize and attack epidermal keratinocytes [17, 18]. This may explain the focal lichenoid dermatitis, intense superficial perivascular inflammation, and epidermal hyperkeratosis/hypergranulosis observed in LPP. The insult on the epidermis could lead to abrupt melanin dropping, which manifests as severe pigmentary incontinence on histopathology. Perifollicular infiltration/fibrosis was found in almost half of the LPP patients in our study. This finding suggests that the lichenoid process often involves the hair follicles and strengthens the well-established relationship between LPP and frontal fibrosing alopecia (FFA). Previous studies have reported that 14%–50% of FFA had preceded LPP [19, 20]. It is possible that patients with perifollicular changes are particularly at risk of developing FFA. However, additional prospective studies are required to prove this. Our study reveals an association with FFA in only 6 LPP patients (8.5%). The condition may be underrecognized due to the retrospective nature of the study, or the incidence may truly be lower in Asian population. As for AD, the pathomechanism is largely unknown, but the focal basal vacuolization and fewer cells indicate an insidious destruction of DEJ, resulting in gradual melanin dropping without much inflammation. The lower degree of inflammation may explain the lower prevalence of pruritus in AD when compared to LPP.

In the past, there had been speculations that LPP are early lesions with inflammatory infiltrates on histopathology while AD presents the late quiescent phase of the same disease. [9, 10, 21]. Al-Mutairi and El-Khalawany reported a significant correlation between the histopathological findings and the duration of the lesion and concluded that there are two histopathological patterns in LPP. Recently developed lesions showed marked vacuolization along the DEJ and band-like lymphocytic infiltration while old lesions had less epidermal changes and mild perivascular infiltration [21]. Subsequent studies then suggested that LPP and AD represent different stages in the evolution of the same pathological process, from lichenoid tissue damage to progressive pigmentary incontinence and melanophage accumulation [9, 10]. However, through reviewing the histopathological changes in relation to the onset of disease, we found no correlation between the inflammatory infiltrate/epidermal changes or melanophage intensity and duration of the rash. We conclude that AD and LPP are 2 different entities. Although both conditions may change over time, distinct histopathological features still persist, particularly the more severe inflammatory infiltrate and pigmentary incontinence in LPP. Additionally, perifollicular infiltration/fibrosis strongly suggests the diagnosis of LPP. In terms of clinicopathological correlations, lichenoid dermatitis and severe pigmentary incontinence both correlate with the purplish-grey colour. As these histopathological features associate with LPP, it is possible that the purplish hue serves as a clinical clue for the diagnosis of LPP.

Our results on their clinical presentations and associations were largely consistent with the previous data. The Thai population is composed of a range of different skin phototypes, but AD and LPP occurred almost exclusively in skin phototypes III-IV. As reported earlier, the apparent female predominance could result from higher cosmetics concerns in females [3]. Associated conditions, including lichen planus, lichen planopilaris, and hepatitis infections, were all recognized in the past. Thyroid disease had been observed in 22% of LPP patients [18], but in our study, hypothyroidism coexisted with AD in 3 patients. Other endocrinopathies, including diabetes mellitus and dyslipidemia, were also reported in LPP by Torres et al. [22]. The relationship between LPP and these endocrine abnormalities were suspected to be caused by the chronic inflammatory state with increases in cytotoxic T-cell activity and proinflammatory cytokines. None of our patients were exposed to antiparasitic agents, fungicides, X-ray, mustard or amla oil, or any other chemicals that were previously linked with AD or LPP [1]. The patterns of hyperpigmentation in LPP were as expected. Recent studies have updated additional variants of LPP, e.g., mimicking discoid lupus erythematosus [23] or a variant on palms and soles [24], none of which was present in our study. From our results, we summarize the clinical and histopathological features of AD and LPP in Table 4.

AD and LPP are both notoriously refractory to treatment [3, 7, 12, 13, 21]. Our results agree that no particular treatment was associated with a significant improvement in the lesion, but the outcome is slightly better in LPP. This finding is consistent with the recent review on treatment outcomes by Wu and Vaidya [25].

The limitations of our study include some missing data due to the retrospective nature. In addition, there was only one biopsy per patient and no follow-up biopsies for assessment of any histopathological changes overtime. Biopsies from multiple sites may have revealed varying severity and allowed a more accurate comparison.

## 5. Conclusions

In conclusion, AD and LPP have distinct clinical presentations. In cases that are not clinically apparent, histopathologic features may help distinguish between these two conditions. Lichenoid dermatitis, epidermal hyperkeratosis/hypergranulosis, perifollicular involvement, and moderate to severe pigmentary incontinence are suggestive findings of LPP, while basal vacuolization along the DEJ and mild pigmentary incontinence are histopathological features favoring AD.

## Figures and Tables

**Table 1:** Baseline characteristics, history, and associated conditions of patients with AD (*n* = 29) and LPP (*n* = 71).

	AD, *n* (%)	LPP, *n* (%)	*p* value
*Baseline characteristics and history of rash*			
Age at diagnosis (years)			
Mean (SD)	42.4 (16.3)	49.6 (13.8)	0.03
Sex			
Male (%)	4 (13.8)	17 (23.9)	0.26
Female (%)	25 (86.2)	54 (76.1)	
Fitzpatrick skin type, *n* (%)			
III	14 (48.3)	26 (36.2)	
IV	14 (48.3)	45 (63.4)	0.13
V	1 (3.5)	0 (0)	
Median rash duration before first visit, weeks (range)	26 (0.42–1,040)	24 (0.42–1,612)	0.69
Pruritus, *n* (%)	4 (13.8)	22 (31.0)	0.08
Burning sensation, *n* (%)	2 (6.9)	3 (4.2)	0.63

*Associated conditions*			
Concurrent lichen planus, *n* (%)	0 (0)	1 (1.4)	1.00
Concurrent lichen planopilaris, *n* (%)	0 (0)	6 (8.5)	0.18
Hypothyroidism, *n* (%)	3 (10.3)	0 (0)	0.02
Hepatitis B infection, *n* (%)	0 (0)	4 (5.6)	0.32
Hepatitis C infection, *n* (%)	0 (0)	2 (2.8)	1.00

**Table 2:** Clinical characteristics of patients with AD (*n* = 29) and LPP (*n* = 71).

Characteristics^*∗*^	AD, *n* (%)	LPP, *n* (%)
Colour		
Slate-grey	17 (58.6)	3 (4.2)
Purplish-grey	2 (6.9)	24 (33.8)
Brown-grey	10 (34.5)	44 (62.0)
Erythematous peripheral rim	1 (3.5)	0 (0)
Morphology		
Macule	7 (24.1)	8 (11.3)
Patch	22 (75.9)	60 (84.5)
Both macules and patches	0 (0)	3 (4.2)
Border		
Well-defined	4 (13.8)	3 (4.2)
Ill-defined	25 (86.2)	68 (95.8)
Pattern of hyperpigmentation		
Diffuse	19 (65.5)	22 (31.0)
Reticular	1 (3.5)	33 (46.5)
Blotchy	9 (31.0)	14 (19.7)
Linear	0 (0)	1 (1.4)
Perifollicular	0 (0)	1 (1.4)
Size of hyperpigmentation		
<1 cm	4 (13.8)	4 (5.6)
1–5 cm	11 (37.9)	38 (53.5)
5–10 cm	12 (41.4)	26 (36.6)
>10 cm	2 (6.9)	3 (4.2)
Distribution		
Sun-exposed area	12 (41.4)	42 (59.2)
Bilateral	24 (82.8)	66 (94.3)
Location		
Face and neck	17 (58.6)	57 (80.3)
Chest	8 (27.6)	10 (14.1)
Back	16 (55.2)	18 (25.4)
Abdomen	14 (48.3)	12 (16.9)
Upper extremities	10 (34.5)	29 (40.9)
Lower extremities	7 (24.1)	17 (23.9)
Flexors (overall)	9 (31.0)	46 (64.8)
(i) Submammary area	1 (3.5)	7 (9.9)
(ii) Axilla	3 (10.3)	8 (11.3)
(iii) Groin	0 (0)	4 (5.6)
(iv) Cubital fossa	2 (6.9)	9 (12.7)
(v) Popliteal fossa	2 (6.9)	8 (11.3)

^*∗*^Clinical characteristics based on recent global consensus [5].

**Table 3:** Histopathological differences between AD (*n* = 29) and LPP (*n* = 71).

Histological features	AD, *n* (%)	LPP, *n* (%)	*p* value
Epidermal atrophy	7 (24.1)	13 (18.3)	0.51
Epidermal hypergranulosis	0 (0)	25 (35.2)	<0.001
Epidermal hyperkeratosis	0 (0)	24 (33.8)	<0.001
Apoptotic keratinocytes	16 (55.2)	47 (66.2)	0.30
Basal vacuolization	27 (96.4)	55 (77.5)	0.02
(i) Focal	27 (100)	51 (92.7)	
(ii) Diffuse	0 (0)	4 (7.3)	
Lichenoid dermatitis	2 (7.1)	35 (49.3)	<0.001
(i) Focal	2 (100)	31 (88.6)	
(ii) Diffuse	0 (0)	4 (11.4)	
Superficial perivascular lymphocytic infiltration	29 (100)	68 (95.8)	0.55
(i) Mild	26 (92.9)	45 (67.2)	0.04
(ii) Moderate	2 (7.1)	19 (28.4)	
(iii) Severe	0 (0)	3 (4.5)	
Deep perivascular lymphocytic infiltration	0 (0)	5 (7.0)	0.32
Perifollicular infiltration	3 (10.3)	34 (47.9)	<0.001
Perifollicular fibrosis	3 (10.3)	25 (35.2)	0.01
Perieccrine infiltration	0 (0)	6 (8.5)	0.18
Pigmentary incontinence			0.015
(i) Mild	3 (10.3)	1 (1.4)	
(ii) Moderate	25 (86.2)	55 (77.5)	
(iii) Severe	1 (3.5)	15 (21.1)	

**Table 4:** Summary of clinical and histopathological features of AD and LPP patients.

	AD	LPP
*Clinical features*		

Gender	Female predominance	Female predominance

Fitzpatrick skin type	Type III-IV	Type III-IV

Pruritus	Less common	More common

Associations	Hypothyroidism	Lichen planus, lichen planopilaris, viral hepatitis

Site	(i) Trunk and proximal extremities	(i) Face and neck, flexural areas, sun-exposed areas
	(ii) Symmetrical distribution	(ii) Symmetrical distribution

Characteristics	(i) Ill-defined slate-grey macules or patches	(i) Ill-defined dark-brown or bluish-brown macules or patches
	(ii) Early lesion may have erythematous rim	(ii) May have different morphologies including diffuse, linear, reticular, follicular, or blotchy
		(iii) May have guttate hypopigmentation

*Histopathologic features*		

Epidermis	(i) Mostly normal	(i) Focal epidermal hyperkeratosis and hypergranulosis
	(ii) Few apoptotic keratinocytes	(ii) Few apoptotic keratinocytes

Basal vacuolization	Present in almost all cases, focal distribution	Present in most cases, can be focal or diffuse

Lichenoid dermatitis	Uncommon	Present in half of the cases, mostly focal

Superficial lymphocytic infiltration	Mild	Moderate to severe

Perifollicular lymphocytic infiltration	None	Present, may develop into perifollicular fibrosis

Pigmentary incontinence	Mild-moderate	Moderate-severe

## Data Availability

The data used to support the findings of this study are available from the corresponding author upon request.
